# Supplementary material for: HIVprotI: an integrated web based platform for prediction and design of HIV proteins inhibitors
Source: J Cheminform. 2018 Mar 9;10:12. doi: 10.1186/s13321-018-0266-y (PMC5845081; doi:10.1186/s13321-018-0266-y)
Supplement: Supplementary file 2 — Additional file 2. Source code of HIVProtI web server. [file 13321_2018_266_MOESM2_ESM.zip › HIVprotI_Source-code/hivprotI/weka-3-6-6/doc/weka/core/RelationalLocator.html]

RelationalLocator


---


|  |  |  |
| --- | --- | --- |
| **Overview** \| **Package** \| **Class** \| **Tree** \| **Deprecated** \| **Index** \| **Help** \| **Weka's home** | |  |
| **PREV CLASS**   **NEXT CLASS** | **FRAMES**    **NO FRAMES**     **All Classes** |  |
| SUMMARY: NESTED \| FIELD \| CONSTR \| METHOD | DETAIL: FIELD \| CONSTR \| METHOD |  |


---


## weka.core Class RelationalLocator

```
java.lang.Object
  weka.core.AttributeLocator
      weka.core.RelationalLocator
```

**All Implemented Interfaces:**: java.io.Serializable, java.lang.Comparable<AttributeLocator>, RevisionHandler

---

``` public class RelationalLocator extends AttributeLocator ```

This class locates and records the indices of relational attributes,

**Version:**
:   $Revision: 6226 $

**Author:**
:   fracpete (fracpete at waikato dot ac dot nz)

**See Also:**: `Attribute.RELATIONAL`, Serialized Form

---

**Constructor Summary**

`RelationalLocator(Instances data)`
  
          Initializes the RelationalLocator with the given data.

`RelationalLocator(Instances data,
int[] indices)`
  
          Initializes the RelationalLocator with the given data.

`RelationalLocator(Instances data,
int fromIndex,
int toIndex)`
  
          Initializes the RelationalLocator with the given data.


| **Method Summary** | |
| --- | --- |
| `static void` | `copyRelationalValues(Instance instance, boolean instSrcCompat, Instances srcDataset, AttributeLocator srcLoc, Instances destDataset, AttributeLocator destLoc)`             Takes relational values referenced by an Instance and copies them from a source dataset to a destination dataset. |
| `static void` | `copyRelationalValues(Instance inst, Instances destDataset, AttributeLocator strAtts)`             Copies relational values contained in the instance copied to a new dataset. |
| `java.lang.String` | `getRevision()`             Returns the revision string. |

**Methods inherited from class weka.core.AttributeLocator**

`compareTo, equals, getActualIndex, getAllowedIndices, getAttributeIndices, getData, getLocator, getLocatorIndices, getType, toString`

**Methods inherited from class java.lang.Object**

`getClass, hashCode, notify, notifyAll, wait, wait, wait`

**Constructor Detail**

### RelationalLocator

```
public RelationalLocator(Instances data)
```

:   Initializes the RelationalLocator with the given data.

    **Parameters:**: `data` - the data to work on

---


### RelationalLocator

```
public RelationalLocator(Instances data,
                         int fromIndex,
                         int toIndex)
```

:   Initializes the RelationalLocator with the given data.
    Checks only the given range.

    **Parameters:**: `data` - the data to work on: `fromIndex` - the first index to inspect (including): `toIndex` - the last index to check (including)

---


### RelationalLocator

```
public RelationalLocator(Instances data,
                         int[] indices)
```

:   Initializes the RelationalLocator with the given data.
    Checks only the specified attribute indices.

    **Parameters:**: `data` - the data to work on: `indices` - the attribute indices to check


**Method Detail**

### copyRelationalValues

```
public static void copyRelationalValues(Instance inst,
                                        Instances destDataset,
                                        AttributeLocator strAtts)
```

:   Copies relational values contained in the instance copied to a new
    dataset. The Instance must already be assigned to a dataset. This
    dataset and the destination dataset must have the same structure.

    :   **Parameters:**: `inst` - the Instance containing the relational values to copy.: `destDataset` - the destination set of Instances: `strAtts` - an AttributeLocator containing the indices of any relational attributes in the dataset.

---


### copyRelationalValues

```
public static void copyRelationalValues(Instance instance,
                                        boolean instSrcCompat,
                                        Instances srcDataset,
                                        AttributeLocator srcLoc,
                                        Instances destDataset,
                                        AttributeLocator destLoc)
```

:   Takes relational values referenced by an Instance and copies them from a
    source dataset to a destination dataset. The instance references are
    updated to be valid for the destination dataset. The instance may have the
    structure (i.e. number and attribute position) of either dataset (this
    affects where references are obtained from). Only works if the number
    of relational attributes is the same in both indices (implicitly these
    relational attributes should be semantically same but just with shifted
    positions).

    :   **Parameters:**: `instance` - the instance containing references to relations in the source dataset that will have references updated to be valid for the destination dataset.: `instSrcCompat` - true if the instance structure is the same as the source, or false if it is the same as the destination (i.e. which of the relational attribute indices contains the correct locations for this instance).: `srcDataset` - the dataset for which the current instance relationvalue references are valid (after any position mapping if needed): `srcLoc` - an AttributeLocator containing the indices of relational attributes in the source datset.: `destDataset` - the dataset for which the current instance relation references need to be inserted (after any position mapping if needed): `destLoc` - an AttributeLocator containing the indices of relational attributes in the destination datset.

---


### getRevision

```
public java.lang.String getRevision()
```

:   Returns the revision string.

    :   **Specified by:**: `getRevision` in interface `RevisionHandler` **Overrides:**: `getRevision` in class `AttributeLocator`
    :   **Returns:**: the revision


---


|  |  |  |
| --- | --- | --- |
| **Overview** \| **Package** \| **Class** \| **Tree** \| **Deprecated** \| **Index** \| **Help** \| **Weka's home** | |  |
| **PREV CLASS**   **NEXT CLASS** | **FRAMES**    **NO FRAMES**     **All Classes** |  |
| SUMMARY: NESTED \| FIELD \| CONSTR \| METHOD | DETAIL: FIELD \| CONSTR \| METHOD |  |


---
